# Phenolic Constituents, Antioxidant and Antimicrobial Activity and Clustering Analysis of Propolis Samples Based on PCA from Different Regions of Anatolia

**DOI:** 10.3390/molecules28031121

**Published:** 2023-01-22

**Authors:** Ümit Altuntaş, İsmail Güzel, Beraat Özçelik

**Affiliations:** 1 Department of Food Engineering, İstanbul Technical University, 34469 İstanbul, Turkey; 2 Department of Food Engineering, Gümüşhane University, 29000 Gümüşhane, Turkey; 3 Department of Mathematical Engineering, İstanbul Technical University, 34469 İstanbul, Turkey; 4 Bioactive Research & Innovation, Food Manuf. Indust. Trade Ltd., Teknokent ARI-3, 34469 Istanbul, Turkey

**Keywords:** propolis, Anatolia, antioxidant activity, phenolics, clustering, PCA

## Abstract

This study aimed to evaluate the biochemical composition and biological activity of propolis samples from different regions of Türkiye to characterize and classify 24 Anatolian propolis samples according to their geographical origin. Chemometric techniques, namely, principal component analysis (PCA) and a hierarchical clustering algorithm (HCA), were applied for the first time to all data, including antioxidant capacity, individual phenolic constituents, and the antimicrobial activity of propolis to reveal the possible clustering of Anatolian propolis samples according to their geographical origin. As a result, the total phenolic content (TPC) of the propolis samples varied from 16.73 to 125.83 mg gallic acid equivalent per gram (GAE/g) sample, while the number of total flavonoids varied from 57.98 to 327.38 mg quercetin equivalent per gram (QE/g) sample. The identified constituents of propolis were phenolic/aromatic acids (chlorogenic acid, caffeic acid, *p*-coumaric acid, ferulic acid, and trans-cinnamic acid), phenolic aldehyde (vanillin), and flavonoids (pinocembrin, kaempferol, pinobanksin, and apigenin). This study has shown that the application of the PCA chemometric method to the biochemical composition and biological activity of propolis allows for the successful clustering of Anatolian propolis samples from different regions of Türkiye, except for samples from the Black Sea region.

## 1. Introduction

Propolis is a complex substance consisting of natural resinous and sticky materials collected by bees, *Apis mellifera*, collected from living plant buds and exudates [1,2,3,4,5,6,7]. Propolis is a wax-like natural product formed by honeybees by combining their own wax with various plant sources [8] and can be considered a non-toxic ‘glue or cement’ for bees [5,8] that provides many benefits to honeybees, such as sealing cracks and plugging holes in the walls of the hive, flattening the inner surface of the hive to minimize moisture loss, regulating humidity and temperature in their nest, and embalming dead insects [1,2,4,9].

The biochemical composition of propolis is quite variable and complex. It depends on factors such as local flora surrounding the hive accessible to honeybees, collection time, geographical origin, type of honeybee, diversity of trees, and plant species collected by honeybees [1,8]. The chemical patterns of propolis types indicate the geographical distribution of plant species [5]. Thus, the chemical composition of propolis differs according to its phytogeographic origin. The variability of colors (yellow, green, brown, and red) of propolis depends on the resin sources found in the region (botanical source), as well as the preparation period [1,2]. However, the chemical composition of some types of propolis, such as European poplar propolis (from *Popolus* spp.), Brazilian green (from *Baccharis dracunculifolia*), and red propolis (from *Dalbergia ecastophyllum*), has been clarified and standardized [4,8].

Propolis consists of about 50% resin (i.e., the polyphenolic fraction, including flavonoids and phenolic/aromatic acids) and 30% wax (waxes and fatty acids), while the essential and aromatic oils (mainly volatiles) and bee-pollen (free amino acids) are approximately 10% of propolis, and the remaining 5% consists of other organic and mineral substances [1,8]. Propolis contains more than 300 natural compounds. A list of pharmacologically active chemical substances reported in several studies are polyphenols (flavonoids, phenolic acids, and their esters), phenolic aldehydes, flavonoid aglycones, sesquiterpenes, quinones, coumarins, amino acids, fatty acids, steroids, terpenoids, and inorganic compounds [1,3,5,7,8,10]. The bioactivity of propolis is variable and is believed to be related to the variation of its chemical composition [8,10]. Due to the presence of bioactive constituents, propolis produces a wide spectrum of important biological activities, such as being antioxidant (strong scavenger of free radicals), antitumor, antibacterial, antifungal, antiviral, anti-inflammatory, cardioprotective, and hepatoprotective, and it has anticancer and immunomodulatory properties [1,3,5,7,8,10,11]. All this has caused propolis to be known as a natural product that can potentially be used as a therapeutic agent to enhance the immune system and prevent various human diseases [1].

The antioxidant activity of propolis protects the human body from cell damage because of free radicals by lowering the number of oxidative chemical reactions that occur [1,3,6,8,12]. Additionally, propolis was recently assessed for its antimicrobial activity against both Gram-positive (+) and -negative (−) bacteria, and previous research has confirmed the in vitro antibacterial activity of propolis extracts via different assays [1,6]. The antioxidant and antibacterial activities of propolis are reported to be caused by flavonoids and phenolic acids and its esters. The interaction and/or synergism between phenolic and other chemicals in propolis is considered the mechanism of this activity [13]. Flavonoids and phenolic compounds are the main bioactive components of propolis [2,3], and both have proven their ability to neutralize free radicals. The wide spectrum of propolis, i.e., biological properties and multiple applications, has aroused interest in the study of its properties according to its origin [2,6].

From the literature reviewed, propolis collected from different places, even from the same city, may differ substantially in terms of antioxidant capacity, antibacterial activity, individual phenolic compounds, and, thus, its biological activities [2,4,5,7,8,10]. Türkiye has a rich phytogeographical structure, and some researchers have attempted to elucidate the chemical structure of propolis produced there. In one of these studies, Kartal et al. (2002) examined propolis samples from the Ankara and Marmaris regions and reported that the botanical origin of the sample from Marmaris could be *Pinus brutia* L. (pine propolis) [14]. In this context, Popova et al. (2005) studied Turkish propolis, and *Populus nigra* and *P. euphratica* were found to be important sources of propolis [15]. Velikova et al. (2000) examined a Bulgarian and two Turkish propolis samples and found that their chemical compositions were similar; they had the characteristics of poplar propolis, and the samples were particularly rich in caffeic acid and ferulic acid [16]. In a study conducted by Sorkun et al. (2001) on Turkish propolis of different geographical origins, it was also found that the samples from Trabzon and Gümüşhane had similar chemical compositions, and the main components were aromatic and aliphatic acids, esters, and ketones. It was found that flavanones, aromatic acid and its esters, terpenoids, flavones, and ketones were the main compounds in the Bursa sample [17]. However, the biochemical data and biological activities of Anatolian propolis are still scarce.

The present study aims to evaluate the biochemical composition and biological activity of propolis samples from different regions of Türkiye to characterize and classify 24 propolis samples based on their geographical origin. In addition, multivariate analysis, principal component analysis (PCA), and a hierarchical clustering algorithm (HCA) were applied to all data, including moisture (%), total phenolic and flavonoid content, antioxidant capacities (DPPH and CUPRAC), individual phenolic and aromatic constituents, and antimicrobial activity to show, for the first time, a possible clustering of Anatolian propolis samples from different regions of origin according to their geographical origin.

## 2. Results

### 2.1. Chemical Properties

The moisture content of the 24 propolis samples belonging to different geographical regions of Türkiye, collected twice in different harvesting seasons, was determined, and the average results are provided in Table 1. According to Table 1, the propolis samples had moisture profiles ranging from 3.83 to 7.13% of total weight. The results of this study are similar to those reported in the literature for other propolis samples, which had moisture content ranging from 3.40 to 9.16% [18,19,20]. Propolis samples from the Marmara region had higher moisture content than other regions. On the other hand, propolis samples from Central Anatolia had relatively lower moisture content. The variations in moisture content (%) of propolis samples are most likely due to the type of propolis, the geographical region and environmental conditions, and the collection period of the propolis [18,21].

### 2.2. Antioxidant Activity

The antioxidant activity of the Turkish propolis samples was determined based on the total phenolic content (TPC) using the Folin–Ciocalteu reagent method, the total flavonoid content (TFC) by indexing the total flavonoids, the cupric ion-reducing antioxidant capacity (CUPRAC) using the copper inducing method, and the scavenging free radical DPPH (2,2-diphenyl-1-picrylhydrazyl) method, since propolis cannot be satisfactorily evaluated by only one method [8,22]. The higher values of the results indicate a high antioxidant capacity for the propolis samples for each method. Table 2 shows the TPC, TFC, and antioxidant capacity (CUPRAC and DPPH) of propolis extract results obtained by analyzing the four different methods mentioned above. Significant differences (*p* < 0.05) in the four different tests between the Anatolian propolis samples are provided in Table 2.

**Figure 1:**
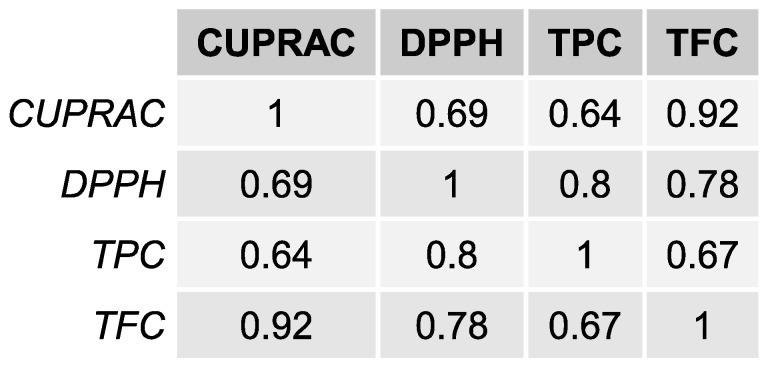
Correlations between the DPPH, CUPRAC, TPC, and TFC results.

The correlation between the method results, DPPH, CUPRAC, TPC, and TFC are shown in Figure 1. A positive, strong correlation is observed between the CUPRAC and TFC (0.92) and between DPPH and TPC (0.80). The lowest correlation was found between the CUPRAC and TPC (0.64).

#### 2.2.1. Total Phenolic Content and DPPH

The Folin–Ciocalteu colorimetric method was used in this study to determine the TPC of Anatolian propolis samples [22]. The TPCs of samples are expressed as gallic acid equivalent (GAE) in 1 g of crude propolis sample. The TPC of the propolis samples from different regions are shown in Table 2. The TPC of the ethanolic extracts of propolis (EEP) samples varied from 16.73 to 125.83 mg GAE/g sample. Based on the region, the highest TPC was found in the Yozgat sample from the Central Anatolia region, whereas the lowest TPC was found in the Gaziantep sample from the Mediterranean region. The lowest TPC values (41.49–16.73 mg GAE/g sample) were found in the samples from the Mediterranean region, while the highest values (125.83–92.51 mg GAE/g sample) were found in the Central Anatolia and Black Sea regions. The TPC values of the samples assayed in this study differed according to the growing region of the cultivars.

The loss of DPPH reagent after the reaction with the samples was measured in the DPPH assay. DPPH is a purple-colored stable radical that turns bright yellow when it combines with free radicals [23]. Propolis’s antioxidant ability was measured in Trolox equivalent (TE) per gram of crude propolis sample. The antioxidant abilities of the EEP samples ranged from 46.72 to 228.23 mg TE/g sample according to the results of the DPPH method. The highest DPPH activity result was observed in the Tokat sample from the Black Sea region, whereas the lowest DPPH activity value was observed in the Gaziantep sample from the Mediterranean region. As can be seen in the TFC profile of the propolis samples, the lowest DPPH activity was obtained in samples from the Mediterranean region (the Hatay, Adana, and Gaziantep samples) and some samples from the Marmara region, namely, İstanbul and Tekirdağ.

The TPC and DPPH revealed a substantial correlation coefficient (*r =* 0.80). These results are in agreement with those of Ahn et al. 2007, who found a good correlation between the antioxidant capacity of the TPC and DPPH (*r* = 0.76) for extracts of Chinese propolis collected throughout China [8,24].

#### 2.2.2. Total Flavonoid Content and CUPRAC

Table 2 represents the TFC of the crude propolis samples; flavonoids varied from 57.98 to 327.38 mg QE per gram of propolis. The highest flavonoid content was found in the Kırklareli sample from the Marmara region, while the lowest value was found in the Gaziantep sample from the Mediterranean region. The lowest TFC was mainly found in the samples from the Mediterranean region, the highest TFC was observed in EEP samples from the Black Sea region of Türkiye, and the relatively lowest was found in the samples from the Marmara region. According to the CUPRAC test results, the propolis samples from the Black Sea region (Zonguldak) showed the highest result (378.93 mg TE/g sample), while the radical scavenging capacity of the Kırklareli sample (370.18 mg TE/g sample) from the Marmara region was slightly higher (Table 2). The CUPRAC results of the samples varied between 61.55 and 378.93 mg TE/g sample. The CUPRAC results of the Mediterranean samples showed the lowest values in this test. The TFC and DPPH results also showed a significant correlation coefficient (r = 0.92), as shown in Figure 1. This was the highest correlation coefficient obtained in these four different measurement tests.

### 2.3. Phenolic Composition of Propolis

Propolis often has a wide range of chemical components. In general, propolis contains flavonoids, phenolic acids and esters, phenolic aldehydes and ketones, terpenes, amino acids, alcohols, and other acids and derivatives [1,25,26]. Several researchers have used HPLC to determine the polyphenolic components of propolis samples, with most applications involving different propolis samples from Europe (Italy), Argentina, and Brazil [27,28]. The individual compounds identified in Anatolian propolis samples are listed in Table 3. Propolis from mild zones (Asia, Europe, North America, etc.) is mostly composed of phenolic compounds, such as various flavonoids and aromatic acids and their esters, obtained from poplar buds, which seem to be the most common source of propolis [29,30]. In our study of 24 Anatolian propolis samples, the identified substances were flavonoids (pinocembrin, kaempferol, pinobanksin, and apigenin), phenolic/aromatic acids (chlorogenic acid, caffeic acid, *p*-coumaric acid, ferulic acid, and trans-cinnamic acid), and phenolic aldehyde (vanillin). Many of these substances (pinobanksin, kaempferol, ferulic acid, caffeic acid, *p*-coumaric acid, etc.) have already been determined by several authors who studied the polyphenolic composition of Turkish [8,17,29] and European propolis samples [31]. Each sample was analyzed in triplicate to determine the average phenolic compound content in Turkish propolis. In our study, ten compounds were identified using the HPLC-PDA system based on ethanolic extracts from propolis samples from four different regions of Anatolia, Türkiye. The peaks of the polyphenolic compounds were detected using a conventional addition procedure and quantified using calibration formulas based on peak normalization rates.

The concentrations of phenolic compounds were calculated as mg/g of the crude propolis sample. The results summarized in Table 3 show that caffeic acid and ferulic acid were the major phenolic acid constituents, while pinobanksin and pinocembrin were the most abundant flavonoids in all propolis samples. Pinobanksin content was lowest in the sample from Gaziantep (2.95 mg/g), which is from the Mediterranean region, and the highest was found in the Ordu sample (38.76 mg/g), which is from the Black Sea region. The other major flavonoid was pinocembrin, whose content ranged from 1.22 mg/g to 6.80 mg/g, with the lowest value found in the Gaziantep sample and the highest value in the Kocaeli sample.

However, in the samples from the Black Sea region, the pinobanksin content was several times higher than the samples from the Marmara and Central Anatolia regions. There is a positive correlation between the number of individual flavonoids and phenolic compounds and their antioxidant capacities [24,31]. The propolis samples that contained more flavonoids and phenols also had higher antioxidant capacities.

### 2.4. Antimicrobial Activity

The disc diffusion method was used to determine the inhibition zones of the 24 different propolis extracts for primary screening. A standard Gram-positive and Gram-negative bacterial strain and two fungi (a yeast and a mold) were used. According to the results shown in Table 4, the EEPs of the samples showed antibacterial activity against *S. aureus* and *E. coli* and antifungal activity against *C. albicans* and *A. niger.* The antibacterial activity of the Anatolian propolis samples tested in this study was effective against the pathogens tested. Table 4 shows that statistically significant differences (*p* < 0.05) were found between Anatolian propolis samples for four different microorganisms.

The antimicrobial activity of propolis has been attributed to both hydrophilic and hydrophobic phenolic chemicals, such as flavonoids and aromatic acids and esters, which can act on the cell walls of bacteria [3]. Caffeic acid and its esters, as well as volatile fractions containing phenols, have antimicrobial properties. However, whether the antibacterial and antifungal activities of the ethanol extracts of propolis depend on the concentration of polyphenolic fractions, such as pinocembrin and caffeic acid derivatives, or on the synergism of these or other compounds is still unknown [29]. Anjum et al. reported that the bioactive compounds of propolis, such as pinocembrin, showed antibacterial activity against *Streptococcus* spp., and artepillin C and *p*-coumaric acid showed antibacterial activity against *Helicobacter pylori* [25].

#### 2.4.1. Antibacterial Activity

Activity against bacterial pathogens was tested for all propolis samples. As can be seen in Table 3, all extracts showed different degrees of antibacterial activity against *S*. *aureus* and *E. coli*, with the strongest antibacterial activity obtained by the samples from Kastamonu, Bartın, and Kırklareli. Among the 24 samples, Sivas, Konya, and Çorum showed moderate activity against *S. aureus*, with inhibition zone values ranging from 8.25 to 9.75 mm. The samples from Kırklareli and Bartın (from the Marmara and Black Sea regions, respectively) showed high activity against *S. aureus* with a 13.5 mm inhibition zone value and against *E. coli* with a 12.5 mm inhibition zone value, respectively. Consequently, the antimicrobial activity test results clearly showed that propolis samples from the Black Sea region had much stronger antibacterial activity compared with the propolis sample from Central Anatolia (Table 3). Previous studies indicated that caffeic acid esters are the main chemicals responsible for this antimicrobial activity [13]. Considering the composition of propolis from Amasya, it had higher caffeic acid content.

#### 2.4.2. Antifungal Activity

The activity of the propolis samples against fungal strains (*C. albicans* and *A. niger*) was tested. All extracts exhibited significant antifungal activity. However, the antifungal activity was similar for all samples. The zones of inhibition around the disc observed in Petri dishes containing *C. albicans* were slightly larger in the Amasya, Mersin, and Adana samples than in the other samples. The highest antifungal activity was obtained in samples from Amasya and Mersin (12.50 mm) for *C. albicans* and from Kırklareli samples (12.50 mm) for the *A. niger* strain. Flavonoids are known for their antibacterial, antifungal, and antiviral capabilities, and they are assumed to be responsible for the beneficial medicinal properties of propolis [30]. Propolis’s antibacterial, antifungal, and antiviral properties have also been observed in the esters of phenolic acids, especially caffeic acids and ferulates [9].

### 2.5. Principal Component Analysis (PCA)

**Figure 2:**
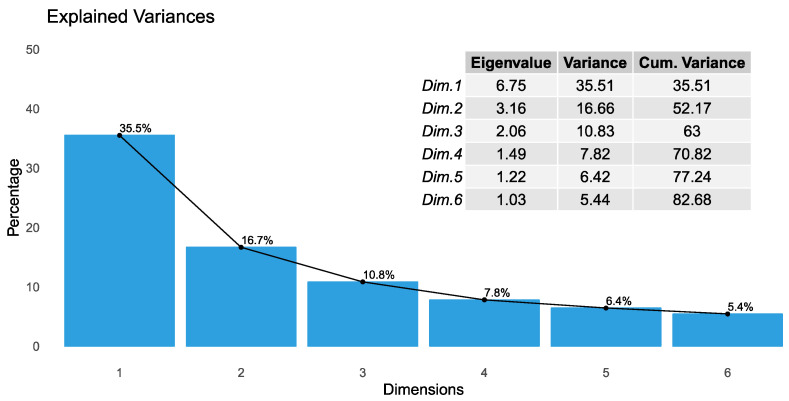
The explained PCs of the total variance and eigenvalues for each of the PCs.

Principal components (PCs) were determined from the eigenvalues of the correlation matrix of observations. The eigenvalues were found to be 6.75, 3.16, 2.06, 1.49, 1.22, and 1.03 for PC1 to PC6, respectively. As can be seen in Figure 2, the first six PCs explain 82.68% of the total variance. The first two PCs account for 35.51% and 16.66% of the total variance, respectively. Thus, the first two PCs explain the origin of the score plot with a very tight confidence ellipse.

**Figure 3:**
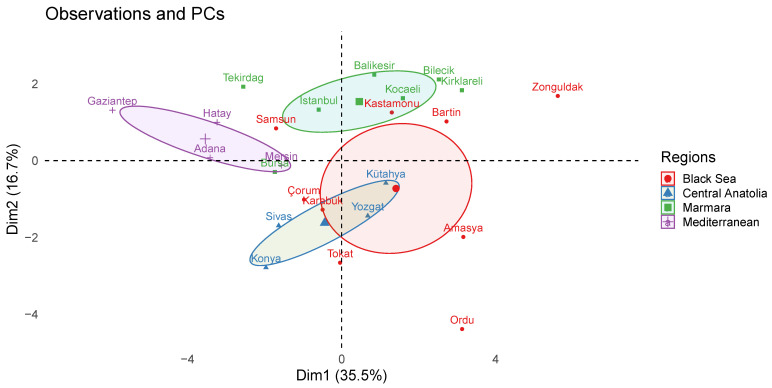
Observations (antimicrobial activity, antioxidant capacity, moisture content (%), and individual phenolic constituents) and PCs belong to Anatolian propolis.

**Figure 4:**
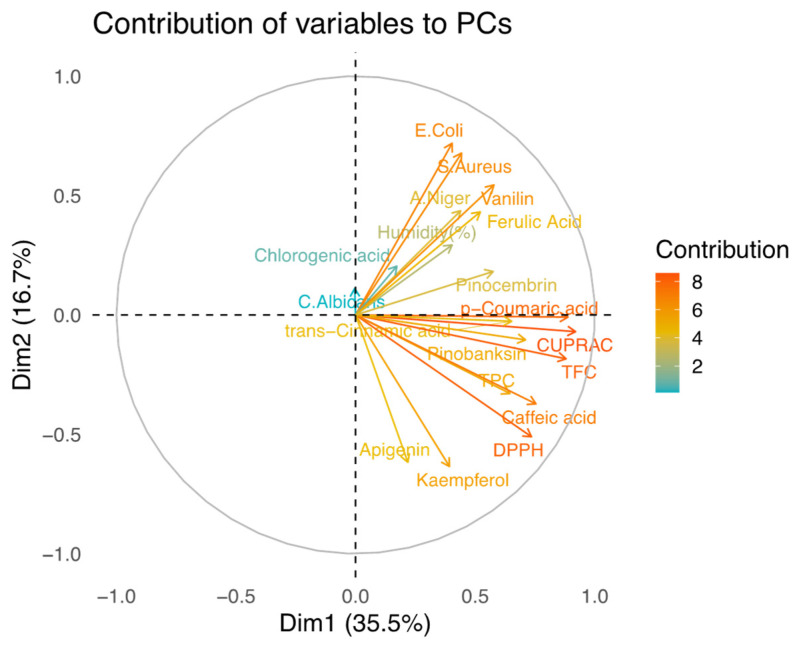
Contribution of variables (TFC, *p*-coumaric acid, DPPH, caffeic acid, etc.) to PCs.

Figure 3 shows the observations and PCs obtained from the analyzed data. The score over PC1 and PC2 separates the groups from the Marmara, Central Anatolia, Mediterranean, and Black Sea regions. The center of the samples from the Marmara region is in the positive parts of both PC1 and PC2, while the samples from Central Anatolia are in the negative parts of both PCs. The Black Sea region samples are grouped along the positive part of PC1, whereas the central point of the samples from the Black Sea region is located on the negative part of PC2. As a result, it can be stated that the DPPH and *E. coli* values as two variables (Figure 4) are useful in clustering the propolis samples from the Marmara and Black Sea regions separately. Mediterranean propolis samples were well separated from the other three groups of samples by being on the negative region of the PC1 due to their significantly higher antifungal activity against *C. albicans*, their relatively low antioxidant capacity determined by four different measurement methods, their low ferulic acid content, and their lack of vanillin content. On the other hand, apart from the *E. coli* and DPPH levels, the *S. aureus*, vanillin, CUPRAC, TFC, and caffeic acid levels played a key role in clustering among the propolis samples from Marmara, the Black Sea, and Central Anatolia.

The contribution of the variables to the PCs is shown in Figure 4. The CUPRAC and *p*-coumaric acid values strongly correlate with PC1 in the positive direction, while the values of *E. coli* and kaempferol correlate with PC2 in the positive and negative directions, respectively. When PCA was applied to map the results of the biochemical composition and biological activity of the propolis samples (*n* = 24), differences in the antioxidant and antimicrobial capacity profiles were observed for samples collected in different parts of Anatolia. This is clearly related to the climatic characteristics and local flora surrounding the hive accessible to honeybees at the collection sites.

**Figure 5:**
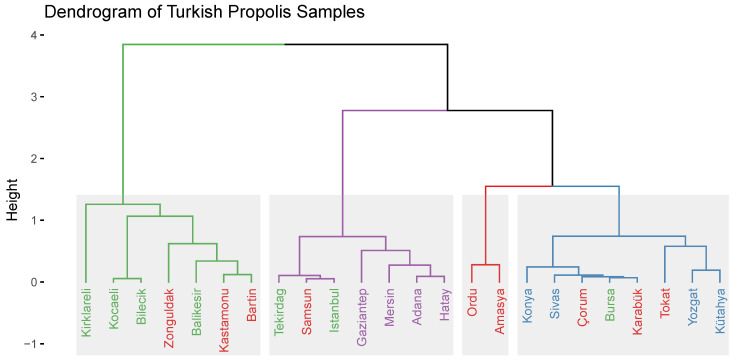
Dendrogram of Turkish propolis samples obtained from the results of HCA by using the factoextra package in R.

A dendrogram of the Turkish propolis samples was generated using a hierarchical clustering algorithm (HCA) with the Ward method using the packages FactoMineR and factoextra in R 4.0.4. This algorithm was applied to the first six PCs since they have eigenvalues greater than 1.0. Figure 5 shows the dendrogram and hierarchical clustering results of the Turkish propolis samples. As can be seen in Figure 5, the propolis samples are divided into four clusters. The Ordu and Amasya samples have a strong relationship with each other and are separated from all the other clusters. Although the Samsun sample belongs to the Black Sea region, it shows a greater similarity with the samples from the Marmara region group. In addition, the samples from the Mediterranean region and two of the samples from the Marmara region (İstanbul and Tekirdağ) are in the same cluster, as shown in Figure 5.

## 3. Discussion

According to the results of the total phenolic content (TPC), total flavonoid content (TFC), and antioxidant capacity measurement methods, phenolic compounds were detected in different amounts and types in the samples from different geographical regions of Türkiye. The antioxidant profile of the propolis samples may be related to the diversity of the geographical areas, plants in the region, and the type of bees [2,32]. Our results for the TPC and DPPH (2,2-diphenyl-1-picrylhydrazyl) methods, remarkably, follow those of Shi et al. (2012) [33], who studied 15 Chinese propolis samples, and Ozdal et al. (2019) [8], who studied 11 Turkish propolis samples. Similarly, the findings of Ahn et al. (2007), who evaluated the TPC of 20 Chinese poplar-type propolis samples, and Yesiltas, who examined 4 Turkish propolis samples, are also quite parallel to our TPC values [24]. However, our TPC results were not as high as those of the samples examined by Yesiltas et al. (2014) [34]. However, the TPC of our samples was relatively higher than the TPC result of Wieczynska et al. (2017) [35], who studied seven Polish propolis samples, and samples of Kubiliene et al. (2015) [36], who obtained the samples under different extraction conditions. Ahn et al. (2007), Moreira et al. (2008) [37], Lagouri et al. (2013) [31], Kumazawa et al. 2004 [33], Ahn et al. (2004) [38], and Shi et al. (2012) reported phenolic compound contents ranging from 42.9 to 329.0 mg/g of the propolis sample in China, Macedonia, Greece, Portugal, and Korea. On the other hand, the results of other studies were reported as IC^50^ units of measurement referring to the prepared solution; this could complicate the comparison of results between different experiments [37]. Alternatively, the results were exressed in mg Trolox or quercetin equivalent per ethanolic propolis extracts with respect to the crude propolis sample [39].

Erdogan et al. (2011) used the DPPH method to assess the antioxidant capabilities of propolis samples from different Anatolian localities, including the cities of Bingol, Rize, Tekirdağ, and Van [40]. According to their results, the highest value for antioxidant activity was obtained in the sample from Rize, with a value of 503.70 mg TE/g sample, which is considerably higher than the DPPH values obtained in our study. The findings of Yesiltas et al. (2014) also showed high DPPH activity values for some of the samples (135–454 TE/g) [34]. Our results for the TFC also follow the TFC results of Shi et al. (2012), Ozdal et al. (2019), and Zarate et al. (2018) [2]. Similarly, the values are also parallel with the results of Ahn et al. (2007) and Yesiltas et al. (2014). However, our findings for the total flavonoid content were higher than the values reported in the studies of Ozdal (2019) and Ahn (2007), respectively. Of the five samples with high TFC values, one sample (Kırklareli) was from the Marmara region, and four of these samples (Amasya, Zonguldak, Ordu, and Tokat) were from the Black Sea region. The surrounding flora and climatic conditions might have contributed to the higher total phenolic and flavonoid content in the propolis samples obtained from these areas. Our results from the cupric ion-reducing antioxidant capacity (CUPRAC) assay are higher than the results (24.00–85.00 TE/g sample) of Ozdal et al. (2019) and slightly lower than the results (575.00 TE/g sample) of Yesiltas et al. (2014).

When the quantitative results of phenols were compared with other publications in the literature (found by Shi et al. (2012), Ahn et al. (2007), Yesiltas et al. (2014), and Pellati et al. (2011)), the number of flavonoids ranged from the lowest value (3.44 mg/g sample) to the highest value (76.50 mg/g sample) for pinobanksin and in a range of 0.43 to 46.00 mg/g sample for pinocembrin [24,33,34,39]. Furthermore, when comparing the quantitative composition of phenolic acids and flavonoids with other studies in the literature, our results for the caffeic acid and *p*-coumaric acid [8,22,29,30,38], ferulic acid [8,22,30,38], and kaempferol [3,29,38] contents were in agreement with the results of these works. Moreover, the ethanolic extracts of our propolis samples were mostly found to be richer in phenolic compounds (the highest values being 5.41, 5.33, and 5.88 mg/g sample for kaempferol, apigenin, and *p*-coumaric acid, respectively) than those of the samples. The caffeic acid content in propolis was reported by Pellati et al. (2011) in Italian poplar-type propolis samples ranging from 0.02 to 1.19 mg/g sample [39]. Ozdal et al. (2019) reported the caffeic acid content in a range between 0.04 and 0.61 mg/g sample in propolis collected from different parts of Türkiye. In our study, we determined the caffeic acid content, ranging from 0.88 to 7.38 mg/g sample. Caffeic acid and *p*-coumaric acid are the most abundant phenolic acids detected in propolis from *Populus* spp. in temperate zones [29]. Most individual phenolic acid and flavonoid contents in 24 Turkish propolis samples are in agreement with the results of other authors [3,8,24,33,34,41]. However, in our study, chlorogenic acid was detected only in the samples from Samsun, Kırklareli, and Hatay. The phenolic content in propolis varies depending on the geographical location; therefore, its biological activity is closely related to biogeographical factors such as local flora, climate, and seasonal influences [26,40]. The variability of the constituents of the propolis samples suggests that some of the propolis samples have different chemical compositions.

Our findings for the antimicrobial activity of Anatolian propolis samples showed that the three propolis extracts were significantly more effective against *S. aureus* than against *E. coli.* These results are in agreement with those reported in the literature [13,42,43], as there is a consistency between the results of all studies showing that propolis extracts are always more efficient against Gram+ than Gram− bacteria. The flavonoids, phenolic acids and esters, and vanillin found in the samples could be responsible for the antibacterial activity against Gram+ bacteria [42,43]. The zones of inhibition against *C. albicans* in the Petri dishes caused by ethanolic extracts (containing 0.1 g crude sample/mL) from Anatolian propolis are in agreement with the results of some authors: 8–14 mm found by Kartal et al. [13], 10.0–12.5 mm found by Silva et al. [44], 13–24 mm found by Aliyazicioglu et al. [45], and 14–18 mm found by Kujumginev et al. [9]. Aliyazicioglu et al. (2013) reported that an inhibitory zone ranging from 8 to 12 mm was obtained against *A. niger*, whereas, in our study, we obtained a zone of inhibition ranging between 8.75 and 12.50 mm [45].

This study showed that by applying chemometric methods, principal component analysis (PCA) on the biochemical composition and biological activity of propolis we were able to successfully group Anatolian propolis samples from different regions of Türkiye, except for the samples from the Black Sea region.

## 4. Materials and Methods

### 4.1. Materials

Folin–Ciocalteu reagent, neocuprine (Nc), 2,2-diphenyl-1-picrylhydrazyl (DPPH), Trolox (≥95%), gallic acid (≥98%), and quercetin (≥95%) were obtained from Sigma-Aldrich. Sodium hydroxide, sodium carbonate, sodium nitrite, potassium persulfate, ferric chloride hexahydrate, ethanol (≥99.8%), methanol (≥99.9%), dipotassium hydrogen phosphate, potassium dihydrogen phosphate, and Whatman^®^ filter papers No. 1 were purchased from Merck (Darmstadt, Germany). Polytetrafluoroethylene (PTFE) filter 0.45 μm, copper (II) chloride, ammonium acetate, aluminum chloride, and potassium chloride were purchased from Fluka Chemie (Buchs, Switzerland). All chemicals used as standards in the HPLC analysis, including apigenin, chlorogenic acid, caffeic acid, ferulic acid, kaempferol, *p*-coumaric acid, pinocembrin, pinobanksin, trans-cinnamic acid, and vanillin, were obtained from Sigma-Aldrich Chemie GmbH (Steinheim, Germany).

#### 4.1.1. Propolis Samples

The Anatolian propolis samples were collected from 4 regions (24 different cities) of Türkiye in June and April 2019. These regions were the Black Sea region (*n* = 9), the Central Anatolia region (*n* = 4), the Marmara region (*n* = 7), and the Mediterranean region (*n* = 4) of Türkiye. All propolis samples were collected from only one provider for each city. The hand-collected propolis samples were ground and pulverized and then stored in individual packages in freezing conditions (−18 °C) until processing for extract preparation. All analyses were performed in triplicate for the 24 propolis samples.

#### 4.1.2. Culture Media and Test Microorganisms

Antimicrobial activities of samples were evaluated against *Staphylococcus aureus*—ATCC 25923—as Gram-positive bacteria, *Escherichia coli*—ATCC 25922—as Gram-negative bacteria, *Candida albicans*—*ATCC 10231*—as yeast, and *Aspergillus niger*—ATCC 16404—as a mold species. All microorganisms were purchased from the ATCC Culture collection and conserved and grown in the Food Engineering Department of Istanbul Technical University laboratories. The screening of antimicrobial activity was performed using Tryptic Soy Agar (TSA, Oxoid Ltd. Hampshire, UK) for bacteria and Sabouraud Dextrose Agar (SDA, Oxoid Ltd., Hampshire, UK) for yeast and fungi.

### 4.2. Methods

#### 4.2.1. Extraction of Propolis

Extraction of propolis was carried out as described by other authors [3,8,46] with some minor modifications. All propolis samples were ground in liquid nitrogen on a lab-scale mill (IKA A11 basic analytical mill, Königwinster, Germany) before extraction. In total, 1 g of the finely ground crude propolis sample was mixed with 10 mL (70%) of aqueous ethanol under constant stirring to obtain a 0.1 g/mL solid extract at room temperature for 24 h. The suspension was then poured into a 50 mL falcon tube and centrifuged at rpm for 10 min to achieve complete separation. After centrifugation, the collected supernatant was filtered through the Whatman filter paper. The filtrate obtained was kept refrigerated and centrifuged again after 24 h at 11,000 rpm at −10 °C for 5 min to remove the wax present in the propolis extract. The final filtrates were kept in the freezer for further analysis. On the other hand, for the microbiological tests, the ethanolic extracts of the propolis samples were dried (first, the ethanol was removed with a rotary evaporator, and then, the aqueous solution was placed in a freeze dryer to remove the water and obtain a dry extract). Then, the dried propolis extracts were dissolved in 10% dimethyl sulfoxide (DMSO) to obtain a final concentration of 0.1 mg/mL dry extract for the microbiological tests.

#### 4.2.2. Determination of Moisture Content

To determine the moisture content of the samples, a halogen infrared moisture analyzer device (HE53, Mettler&Toledo, Zaventem, Belgium) was used. On a tared measuring cup made of aluminum foil, the previously powdered propolis samples were weighed as 0.5 g and placed in the instrument, and the moisture content of the samples was determined by reading the value directly on the screen. All measurements were performed in triplicate.

#### 4.2.3. Antimicrobial Activity Test

The antibacterial and antifungal activity of propolis samples was assayed by using the disc diffusion method on agar [47]. The antibacterial activity of propolis was tested using TSA for bacteria and using SDA (Oxoid) as inoculum for the antifungal activity of propolis samples. *S. aureus*, *E. coli*, *C. albicans*, and *A. niger* strains were used as test microorganisms. The antimicrobial activity was determined by calculating the diameter of the inhibitory zones in a Petri dish, which were discolored after 24 h of incubation at 37 °C. An inhibitory zone with a diameter of less than 5 mm was considered inactive (the diameter of the spot was 5 mm) [9]. The control experiments showed that the solvent used as a control had no activity. All microorganisms used as inoculum were cultured overnight at 37 °C in TSB. The turbidity of these suspensions was adjusted to approximately 1 × 10^6^ CFU/mL by dilution with peptone water. Colonized agar Petri dishes were prepared (15–20 mL) and inoculated with 100μL of suspensions containing approximately 10 microorganisms per ml. Then, sterile paper discs (diameter = 6 mm) were positioned on the agar to load 100 μL of each propolis extract (a concentration of 0.1 mg propolis extract in 1 mL of 10% DMSO). All propolis extracts were dissolved in the 10% DMSO prepared previously. In addition, DMSO (10%) was used as a negative control, and commercial discs of Ampicillin (10 mg—Oxoid) were used as a positive control for antibacterial activity, while commercial discs of vorozanole (11 mg—Oxoid) were used as a positive control for antifungal activity. *S. aureus* and *E. coli* strains were incubated at 37 °C for 24 h, *C. albicans* at 37 °C for 48 h, and *A. niger* at 25 °C for 72 h. Subsequently, the zones of inhibition around the discs were determined in millimeters (mm). All test results were triple-checked.

#### 4.2.4. Total Flavonoid and Phenolic Content and Antioxidant Activity Tests

##### Determination of Total Flavonoid Content

The total flavonoid content (TFC) of the propolis samples was determined by reading a prepared mixture in a spectrophotometer, as described by Kim, Jeong, and Lee (2003) [48] and modified by Uluata et al. [49] (2021) and Kızıltaş (2021) [50]. The absorbance of the mixture was measured at 510 nm. All test results were triplicated. A quercetin standard curve was prepared to determine the TFC of the extracts, and the results were expressed as mg quercetin equivalent (QE) per gram of crude sample.

##### Determination of Total Phenolic Content

The total phenolic content (TPC) of propolis was determined using the Folin–Ciocalteu method, as described by Chen et al. 2015, Hızır-Kadı et al. 2020, and Topal et al. 2021 [51,52,53]. After 45 min of storage at room temperature in a dark place, the absorbance of the mixtures was measured at 765 nm using a Biotek Synergy HTC multimode microplate reading spectrophotometer (Biotek Instruments Inc., USA). Phenolic content was calculated using a standard curve generated with gallic acid. All measurements were performed in triplicate. TPC concentration was expressed as mg gallic acid equivalent (GAE) per gram of crude sample.

##### Determination of DPPH Activity

The DPPH assay was also used to determine the radical scavenging power of the propolis samples according to the method described by Apak et al. 2014 [54]. The violet free radical DPPH was measured at 517 nm to determine decolorization. All measurements were performed in triplicate. Trolox was used as a standard, and results were expressed as μg Trolox equivalent (TE) per gram of crude sample.

##### Determination of Cupric Ion-Reducing Antioxidant Capacity (CUPRAC)

The CUPRAC assay was performed on propolis samples according to Apak et al., 2004 [54], modified by Pasli et al., 2019 [55]. CuCl_2_ solution, 10^−2^ mM, was prepared in distilled water. In total, 19.27 g of NH_4_Ac was dissolved in distilled water and diluted to 250 mL to prepare an ammonium acetate buffer with a pH of 7.0 and a concentration of 1.0 M. A neocuproine (Nc) solution in ethanol (7.5 × 10^−3^ M) was freshly prepared. In total, 1 mL of the CuCl_2_ solution, Nc solution, ammonium acetate buffer, and distilled water were added to 100 μL of the extract to provide a total of 4.1 mL of the mixture. After 30 min, absorbance was measured at 450 nm against a reagent blank. All measurements were performed in triplicate. Trolox was used as a standard, and results were expressed as mg TE per gram of crude sample.

#### 4.2.5. Sample Preparation for HPLC Analysis

After completing the extraction, an aliquot extract (1 mL) was evaporated to dryness in a rotary evaporator (IKA RV10, Germany) at 40 °C. After evaporation, the solvent was dissolved in 10 mL of pure methanol. It was diluted 100-fold with MeOH and filtered through a 0.45 μm PTFE filter (Waters, Milford, CA, USA) immediately before injection into the HPLC system. All sample preparations were performed in triplicate; thus, quantification data are the average of the three results.

#### 4.2.6. HPLC-PDA Analysis

For HPLC analyses, propolis samples at a concentration of 1 mg/mL were injected into a Shimadzu 20A Series ultrafast liquid chromatograph (UFLC, Shimadzu Corporation, Japan) equipped with a microvacuum degasser, autosampler, column oven, controller, and PDA detector. An ACE C18 column (250 mm × 4,6 mm, 3 μm) was used for chromatographic separations. LC Solution software (Shimadzu Corporation, Kyoto, Japan) was used for data acquisition and elaboration. The mobile phases were water with 0.75% formic acid (*v*/*v*) (solvent A) and HPLC-grade methanol with 0.75% formic acid (*v*/*v*) (solvent B). A gradient of mobile phase A and mobile phase B was used with a flowrate of 0.5 mL and 10 μL of injection volume for each standard mixture, and the column temperature was set at 40 °C [48]. The mobile phase was degassed in an ultrasonic bath and filtered through a 0.45 μm PTFE filter before use. A blank injection was also used to assess chromatographic interference at resolution. Stock solutions of the chemical standards were prepared in a final volume of 10 mL MeOH at a concentration of 1000 μg/mL. Each standard’s stock solutions were prepared in methanol at 10 mg/mL and kept at −20 °C. The working standard solutions were prepared at 5 calibration levels with final concentrations of 6.25, 12.5, 25, 50, and 100 g/mL. The phenolic/aromatic acids, phenolic aldehyde, and flavonoids present in the samples were identified and quantified by comparing the retention time and the size of the peaks in the methanolic extracts with those of the standard components as follows: chlorogenic acid, caffeic acid, vanillin, apigenin, kaempferol, *p*-coumaric acid, ferulic acid, trans-cinnamic acid, pinobanksin, and pinocembrin.

#### 4.2.7. Statistical Analysis

All statistical analyses of the data obtained were evaluated using R Statistical Program version 4.0.4 (R Core Team) and MINITAB Statistical Program version 19 (Minitab Inc.). One-way ANOVA and Tukey’s post hoc test for multiple comparisons with statistical significance at a 95% confidence level (*p* < 0.05) were performed to identify group differences. Analytical data from Anatolian propolis samples were subjected to principal component analysis (PCA), multivariate statistical analysis [56]. Pearson’s correlation test was used to determine the correlation between antioxidant activities (CUPRAC, DPPH) with TPC and TFC by using R 4.0.4.

Recent studies have revealed that variables such as antioxidants and antimicrobial activities, individual phenolic and flavonoid substances of bee products are significant, with the greatest discriminatory power in a PCA [4,57,58]. Therefore, these variables were selected to accurately group the Anatolian propolis samples according to their geographical origin.

## 5. Conclusions

This work clarified the phenolic composition and antioxidant and antimicrobial activities of 24 Anatolian propolis samples from Türkiye. The current study revealed the presence of phytochemicals, mainly caffeic acid, *p*-coumaric acid, ferulic acid, pinobanksin, and apigenin in Turkish propolis samples. We conclude that ethanolic extracts of propolis could be a useful adjunct to pharmaceutical products in improving human health by aiding the antioxidant defense system in combating free radical formation. Our results clearly demonstrate that Anatolian propolis samples have remarkable antioxidant and antimicrobial activities, which was expected since propolis is considered the bee’s defense system against infections.

Our results and the data from the literature on propolis’s chemical composition and biological action do not point to a single compound or class of compounds that could be responsible for this effect. The biochemical properties of Anatolian propolis appear to have broad therapeutic significance as a natural mixture rather than as a source of a novel antibacterial, antifungal, or antiviral chemical. The samples were well clustered using principal component analysis, with antioxidant and antimicrobial activity and phenolic compound values as parameters. The first two principal components were used to separate the Turkish propolis samples from each other and proved to be an efficient way to classify the propolis samples into groups according to the collection site.

## Figures and Tables

**Table 1:** Moisture content (%) of Anatolian propolis samples (data are expressed as g water/100 g of the crude sample).

Region	City	Moisture Content (%)
Black Sea	Amasya	5.12 ± 0.1 ^g^
Black Sea	Bartın	5.88 ± 0.1 ^b,c^
Black Sea	Karabük	5.05 ± 0.1 ^d,e^
Black Sea	Kastamonu	4.52 ± 0.3 ^e,f^
Black Sea	Ordu	5.09 ± 0.1 ^d,e^
Black Sea	Samsun	5.22 ± 0.1 ^c,d,e^
Black Sea	Tokat	6.25 ± 0.1 ^a,b^
Black Sea	Zonguldak	5.35 ± 0.0 ^c,d^
Black Sea	Çorum	4.01 ± 0.1 ^f,g^
Central Anatolia	Konya	3.84 ± 0.1 ^g^
Central Anatolia	Kütahya	5.15 ± 0.1 ^c,d,e^
Central Anatolia	Sivas	3.83 ± 0.1 ^g^
Central Anatolia	Yozgat	4.24 ± 0.1 ^e,f^
Marmara	Balıkesir	7.13 ± 0.3 ^a^
Marmara	Bilecik	6.07 ± 0.1 ^b,c^
Marmara	Bursa	5.72 ± 0.3 ^b,c^
Marmara	İstanbul	6.22 ± 0.1 ^a,b^
Marmara	Kırklareli	5.26 ± 0.5 ^c,d,e^
Marmara	Kocaeli	5.22 ± 0.1 ^c,d,e^
Marmara	Tekirdağ	4.17 ± 0.1 ^c,d,e^
Mediterranean	Adana	4.61 ± 0.1 ^e,f^
Mediterranean	Gaziantep	3.70 ± 0.1 ^g^
Mediterranean	Hatay	5.86 ± 0.1 ^b,c^
Mediterranean	Mersin	3.83 ± 0.1 ^g^

Significant differences in the same column are represented by different letters (a–g) (*p* < 0.05).

**Table 2:** Total phenolic and flavonoid contents and antioxidant capacity results (CUPRAC and DPPH) of ethanolic extracts of Anatolian propolis samples (data are expressed as mg/g of the crude propolis sample).

Region	City	CUPRAC (mg TE/g Sample)	DPPH (mg TE/g Sample)	TPC (mg GAE/g Sample)	TFC (mg QE/g Sample)
Black Sea	Amasya	345.60 ± 6.7 ^a,b,c^	186.84 ± 8.0 ^a,b^	88.32 ± 1.1 ^b,c^	325.09 ± 11.3 ^a^
Black Sea	Bartın	328.23 ± 37.1 ^a,b,c^	157.58 ± 9.8 ^b,c,d^	92.51 ± 3.1 ^b^	251.47 ± 56.3 ^a,b,c^
Black Sea	Karabük	257.43 ± 28.4 ^c,d,e^	160.94 ± 30.5 ^b,c,d^	66.42 ± 8.9 ^d,e,f^	240.28 ± 38.2 ^b,c,d^
Black Sea	Kastamonu	297.36 ± 39.0 ^b,c^	166.81 ± 23.4 ^a,b,c^	76.51 ± 8.0 ^c,d^	237.07 ± 32.9 ^c,d,e^
Black Sea	Ordu	357.73 ± 9.4 ^a,b^	214.50 ± 22.3 ^a,b^	70.23 ± 8.2 ^c,d,e^	301.71 ± 47.8 ^a,b^
Black Sea	Samsun	196.35 ± 38.7 ^e,f,g^	128.07 ± 7.3 ^d,e,f^	70.26 ± 2.2 ^c,d,e^	222.04 ± 8.8 ^c,d,e^
Black Sea	Tokat	233.45 ± 20.0 ^c,d,e^	228.23 ± 30.1 ^a^	96.00 ± 8.2 ^b^	289.68 ± 12.3 ^a,b,c^
Black Sea	Zonguldak	378.93 ± 29.7 ^a^	175.00 ± 12.0 ^a,b^	98.89 ± 5.3 ^b^	323.71 ± 9.4 ^a^
Black Sea	Çorum	243.99 ± 16.2 ^c,d,e^	151.82 ± 8.3 ^c,d^	81.33 ± 3.3 ^c,d^	217.56 ± 19.8 ^c,d,e^
Central Anatolia	Konya	127.40 ± 6.5 ^g,h,i^	164.41 ± 4.0 ^a,b,c^	110.37 ± 5.0 ^a,b^	147.04 ± 3.0 f^g^
Central Anatolia	Kütahya	360.93 ± 46.8 ^a,b^	160.82 ± 7.6 ^b,c,d^	88.44 ± 13.2 ^b,c^	283.26 ± 37.1 ^a,b,c^
Central Anatolia	Sivas	173.21 ± 17.2 ^f,g^	143.26 ± 10,1 ^c,d^	56.89 ± 8.8 ^e,f^	167.73 ± 33.9 ^e,f,g^
Central Anatolia	Yozgat	304.88 ± 12.0 ^b,c^	195.36 ± 47.4 ^a,b^	125.83 ± 24.0 ^a^	241.04 30.9 ^b,c,d^
Marmara	Balıkesir	225.34 ± 21.6 ^d,e,f^	167.32 ± 6.2 ^a,b,c^	68.88 ± 10.3 ^d,e^	208.50 ± 4.0 ^d,e^
Marmara	Bilecik	259.75 ± 30.8 ^c,d,e^	169.74 ± 4.7 ^a,b,c^	71.14 ± 1.8 ^c,d,e^	250.87 ± 16.1 ^a,b,c^
Marmara	Bursa	200.73 ± 22.8 ^e,f,g^	143.14 ± 38.2 ^c,d,e^	51.90 ± 9.6 ^e,f^	150.46 ± 22.4 ^f,g^
Marmara	İstanbul	227.44 ± 13.7 d^e,f^	111.48 ± 20.6 ^d,e,f^	55.69 ± 16.5 ^e,f^	189.54 ± 35.5 ^e,f,g^
Marmara	Kırklareli	370.18 ± 31.6 ^a^	157.61 ± 3.6 ^b,c,d^	83.20 ± 9.1 ^c^	327.38 ± 21.6 ^a^
Marmara	Tekirdağ	101.36 ± 4.0 ^h,i^	90.46 ± 3.9 ^e,f^	48.49 ± 5.3 ^e,f^	91.78 ± 4.8 ^g^
Marmara	Kocaeli	262.29 ± 28.0 ^b,c,d^	138.26 ± 1.1 ^d,e,f^	71.51 ± 6.8 ^c,d^	182.61 ± 15.0 ^e,f,g^
Mediterranean	Adana	147.95 ± 40.9 ^g,h^	80.58 ± 10.9 ^e,f^	41.91 ± 8.8 ^e,f^	142.70 ± 27.4 ^f,g^
Mediterranean	Gaziantep	61.55 ± 3.0 ^h^	46.72 ± 2.1 ^f^	16.73 ± 1.0 ^f^	57.98 ± 1.0 ^g^
Mediterranean	Hatay	122.04 ± 5.7 ^g,h,i^	85.72 ± 4.6 ^e,f^	34.17 ± 2.9 ^e,f^	109.07 ± 2.9 ^f,g^
Mediterranean	Mersin	151.79 ± 21.7 ^f,g,h^	111.74 ± 11.9 ^d,e,f^	41.38 ± 6.6 ^e,f^	167.37 ± 6.6 ^e,f,g^

Significant differences in the same column are represented by different letters (a–h) (*p* < 0.05). TE (Trolox equivalent); GAE (gallic acid equivalent); QE (quercetin equivalent).

**Table 3:** Content of individual compounds (phenolic acids, phenolic aldehyde, and flavonoids) determined in Anatolian propolis samples (data are expressed as mg/g of the crude propolis sample).

City	Chlorogenic Acid	Caffeic Acid	Vanillin	*p*-Coumaric Acid	Ferulic Acid	trans-Cinnamic Acid	Pinobanksin	Kaempferol	Apigenin	Pinocembrin
Amasya	-	7.17 ± 2.6 ^a^	0.48 ± 0.4 ^c^	2.80 ± 0.6 ^c^	3.09 ± 1.6 ^c,d^	7.84 ± 0.3 ^a^	19.55 ± 7.3 ^b^	5.41 ± 1.4 ^a,b^	3.78 ± 0.1 ^a,b^	2.41 ± 0.2 ^b,c^
Bartın	-	4.67 ± 2.0 ^b,c^	0.83 ± 0.1 ^b^	3.95 ± 0.9 ^b^	5.17 ± 0.1 ^c^	4.65 ± 0.1 ^b^	21.53 ± 3.2 ^b^	4.06 ± 1.2 ^b^	1.73 ± 0.1	2.24 ± 0.2 ^b,c^
Karabük	-	3.42 ± 1.2 ^d,e^	0.32 ± 0.1 ^c,d^	2.21 ± 0.9 ^c,d^	0.83 ± 0.2 ^e^	1.37 ± 0.8 ^e^	10.59 ± 3.1 ^c^	4.30 ± 1.4 ^b^	2.79 ± 0.1 ^b^	3.41 ± 0.8 ^b^
Kastamonu	-	4.42 ± 0.8 ^c^	0.46 ± 0.3 ^c^	3.64 ± 1.6 ^b^	4.21 ± 3.4 ^c,d^	0.51 ± 0.3 ^e,f^	34.66 ± 0.7 ^a^	1.48 ± 0.1 ^c,d^	0.43 ± 0.1 ^c,e^	2.92 ± 0.7 ^b,c^
Ordu	-	7.38 ± 3.5 ^a^	-	4.43 ± 1.9 ^a,b^	1.78 ± 1.6 ^d,e^	2.93 ± 1.5 ^c,d^	38.76 ± 1.5 ^a^	6.95 ± 1.5 ^a^	4.10 ± 1.0 ^a,b^	3.86 ± 1.0 ^b^
Samsun	0.31 ± 0.1 ^c^	1.93 ± 0.9 ^e,f^	0.18 ± 0.1 ^d^	1.11 ± 0.1 ^d^	2.05 ± 1.2 ^d^	0.97 ± 0.5 ^e,f^	13.75 ± 0.6 ^b,c^	1.73 ± 0.6 ^c^	1.41 ± 0.3 ^b,c^	2.09 ± 1.0 ^c^
Tokat	-	3.44 ± 0.5 ^d,e^	-	2.25 ± 0.5 ^c,d^	2.59 ± 0.2 ^d^	0.53 ± 0.4 ^e,f^	8.15 ± 3.2 ^c,d^	2.07 ± 0.2 ^c^	5.33 ± 1.6 ^a^	1.48 ± 0.2 ^c^
Zonguldak	-	6.62 ± 2.8 ^a,b^	1.37 ± 0.9 ^c^	5.86 ± 3.3 ^a^	10.77 ± 1.8 ^b^	7.45 ± 0.3 ^a^	26.65 ± 2.2 ^a,b^	2.95 ± 1.2 ^b,c^	0.87 ± 0.1 ^c^	6.53 ± 2.4 ^a^
Çorum	-	3.56 ± 0.7 ^d,e^	0.14 ± 0.1 ^d^	1.57 ± 0.1 ^d^	1.54 ± 0.3 ^d,e^	0.72 ± 0.2 ^e,f^	16.18 ± 0.2 ^b^	2.12 ± 0.1 ^c^	1.75 ± 0.4 ^b,c^	5.25 ± 0.3 ^a,b^
Konya	-	4.64 ± 0.1 ^c^	0.27 ± 0.1 ^c,d^	2.14 ± 0.1 ^c,d^	0.94 ± 0.1 ^e^	0.86 ± 0.1 ^e,f^	12.37 ± 4.0 ^b,c^	2.85 ± 0.1 ^b,c^	1.84 ± 0.1 ^b,c^	3.17 ± 1.1 ^b^
Kütahya	-	4.12 ± 1.3 ^c,d^	-	2.75 ± 0.9 ^c^	1.68 ± 0.2 ^d,e^	4.02 ± 0.1 ^b^	17.30 ± 0.2 ^b^	2.61 ± 0.1 ^b,c^	1.55 ± 0.4 ^b,c^	2.42 ± 0.1 ^b,c^
Sivas	-	5.28 ± 0.1 ^b^	0.42 ± 0.1 ^c^	2.63 ± 0.3 ^c^	2.65 ± 0.4 ^d^	1.01 ± 0.1 ^e^	10.21 ± 1.6 ^c^	4.39 ± 0.1 ^b^	1.50 ± 0.1 ^b,c^	2.82 ± 0.1 ^b,c^
Yozgat	-	3.64 ± 2.0 ^d,e^	0.65 ± 0.2 ^b,c^	2.57 ± 0.3 ^c^	5.50 ± 0.5 ^c^	0.94 ± 0.1 ^e,f^	13.39 ± 2.8 ^b,c^	3.90 ± 0.4 ^b^	1.52 ± 0.1 ^b,c^	2.31 ± 0.7 ^b,c^
Balıkesir	-	4.46 ± 1.9 ^c^	0.61 ± 0.4 ^b,c^	3.23 ± 2.3 ^b,c^	2.24 ± 0.5 ^d^	2.86 ± 0.7 ^d^	18.02 ± 3.1 ^b^	-	1.76 ± 0.4 ^b,c^	2.03 ± 1.6 ^c^
Bilecik	-	3.98 ± 0.9 ^c,d^	1.54 ± 1.2 ^a^	3.83 ± 2.2 ^b^	17.28 ± 2, 4 ^a^	1.63 ± 0.3 ^e^	16.19 ± 2.1 ^b^	3.04 ± 1.2 ^b,c^	1.48 ± 0.2 ^b,c^	6.57 ± 1.6 ^a^
Bursa	-	4.36 ± 1.9 ^c^	0.57 ± 0.1 ^b,c^	2.53 ± 1.5 ^c^	1.27 ± 0.1 ^d,e^	0.79 ± 0.1 ^e,f^	10.79 ± 2.4 ^c^	1.72 ± 0.4 ^c^	1.30 ± 0.3 ^b,c^	2.55 ± 0.1 ^b,c^
İstanbul	-	3.24 ± 1.3 ^e^	0.74 ± 0.1 ^b^	2.10 ± 0.1 ^c,d^	5.67 ± 0.3 ^c^	3.49 ± 0.3 ^b,c^	10.93 ± 6.0 ^c^	1.89 ± 0.1 ^c^	1.84 ± 0.5 ^b,c^	2.54 ± 0.1 ^b,c^
Kırklareli	1.56 ± 0.1 ^a^	5.78 ± 2.5 ^b^	0.97 ± 0.8 ^b^	2.51 ± 1.6 ^c^	2.75 ± 0.2 ^d^	1.60 ± 0.2 ^e^	20.74 ± 5.6 ^b^	1.75 ± 0.1 ^c^	2.31 ± 0.1 ^b^	5.35 ± 2.1 ^a,b^
Kocaeli	-	3.69 ± 1.9 ^d,e^	1.56 ± 1.3 ^a^	3.93 ± 2.1 ^b^	14.59 ± 2.2 ^a^	-	16.82 ± 1.4 ^b^	4.00 ± 1.6 ^b^	1.91 ± 0.1 ^b,c^	6.80 ± 0.3 ^a^
Tekirdağ	-	1.09 ± 0.1 ^f^	0.74 ± 0.1 ^b^	1.11 ± 0.2	5.10 ± 0.7 ^c^	0.45 ± 0.1 ^f^	10.01 ± 3.3 ^c^	1.01 ± 0.1 ^d^	1.12 ± 0.1 ^b,c^	1.44 ± 0.1 ^c^
Adana	-	2.24 ± 0.4 ^e,f^	-	1.19 ± 0.5 ^d^	0.93 ± 0.1 ^e^	1.68 ± 0.3 ^e^	9.11 ± 3.5 ^c,d^	2.58 ± 0.1 ^b,c^	1.80 ± 0.1 ^c^	1.68 ± 0.4 ^c^
G.antep	-	0.88 ± 0.1 ^f^	-	0.20 ± 0.1 ^f^	0.45 ± 0.1 ^f^	0.24 ± 0.1 ^f,g^	2.95 ± 0.1 ^d^	1.86 ± 0.1 ^c^	0.41 ± 0.1 ^c,e^	1.22 ± 0.1 ^c^
Hatay	0.69 ± 0.1 ^b^	3.75 ± 2.5 ^d^	-	0.51 ± 0.2 ^e^	0.41 ± 0.1 ^f^	0.56 ± 0.1 ^e,f^	11.44 ± 1.5 ^c^	1.91 ± 0.1 ^c^	1.56 ± 0.5 ^b,c^	1.99 ± 0.1 ^c^
Mersin	-	4.62 ± 2.9 ^c^	-	1.17 ± 0.2 ^d^	0.83 ± 0.1 ^e^	0.32 ± 0.1 ^f^	21.70 ± 3.3 ^b^	2.88 ± 0.1 ^b,c^	3.82 ± 0.5 ^a,b^	4.25 ± 0.1 ^b^

Significant differences in the same column are represented by different letters (a–f) (*p* < 0.05).

**Table 4:** Antimicrobial activity results of ethanolic extracts of Anatolian propolis (data are expressed as millimeter (mm) inhibition zone).

Region	City	*S. aureus*	*E. coli*	*C. albicans*	*A. niger*
Black Sea	Amasya	12.00 ± 1.0 ^a,b^	10.50 ± 0.5 ^a,b^	12.50 ± 1.5 ^a,b^	9.50 ± 0.5 ^a,b^
Black Sea	Bartın	12.50 ± 1.5 ^a,b^	12.50 ± 1.5 ^a^	10.00 ± 1.0 ^a,b^	11.00 ± 1.0 ^a,b^
Black Sea	Karabük	12.00 ± 1.0 ^a,b^	9.50 ± 1.5 ^a,b^	10.50 ± 0.5 ^a,b^	8.75 ± 0.3 ^b,c^
Black Sea	Kastamonu	14.00 ± 1.0 ^a^	11.50 ± 0.5 ^a,b^	10.50 ± 0.5 ^a,b^	9.50 ± 0.5 ^a,b^
Black Sea	Ordu	10.25 ± 0.2 ^a,b^	10.50 ± 0.5 ^a,b^	9.00 ± 0.0 ^b^	9.00 ± 1.0 ^b,c^
Black Sea	Samsun	11.25 ± 1.2 ^a,b^	11.50 ± 0.5 ^a,b^	10.00 ± 1.0 ^a,b^	9.50 ± 0.5 ^a,b^
Black Sea	Tokat	9.75 ± 0.7 ^a,b^	9.00 ± 0.0 ^a,b^	10.50 ± 0.5 ^a,b^	10.00 ± 1.0 ^a,b^
Black Sea	Zonguldak	13.50 ± 0.5 ^a,b^	11.50 ± 0.5 ^a,b^	11.00 ± 1.0 ^a,b^	11.00 ± 0.0 ^a,b^
Black Sea	Çorum	9.25 ± 0.7 ^a,b^	9.50 ± 0.5 ^a,b^	10.50 ± 0.5 ^a,b^	10.00 ± 0.0 ^a,b^
Central Anatolia	Konya	9.00 ± 0.2 ^a,b^	8.00 ± 0.0 ^b^	9.00 ± 1.0 ^b^	8.00 ± 0.0 ^c^
Central Anatolia	Kütahya	11.25 ± 1.2 ^a,b^	9.50 ± 0.5 ^a,b^	9.50 ± 0.5 ^a,b^	12.00 ± 1.0 ^a^
Central Anatolia	Sivas	8.25 ± 0.7 ^b^	9.50 ± 0.5 ^a,b^	11.50 ± 1.5 ^a,b^	9.00 ± 1.0 ^b,c^
Central Anatolia	Yozgat	11.00 ± 1.0 ^a,b^	9.50 ± 0.5 ^a,b^	9.00 ± 0.0 ^b^	9.50 ± 0.5 ^a,b^
Marmara	Balıkesir	13.50 ± 1.5 ^a,b^	12.00 ± 1.0 ^a,b^	12.00 ± 1.0 ^a,b^	10.50 ± 0.5 ^a,b^
Marmara	Bilecik	12.50 ± 1.5 ^a,b^	11.00 ± 1.0 ^a,b^	10.00 ± 0.0 ^a,b^	9.50 ± 0.5 ^a,b^
Marmara	Bursa	11.00 ± 1.0 ^b^	8.75 ± 0.3 ^a,b^	10.00 ± 1.0 ^a,b^	8.50 ± 1.0 ^b,c^
Marmara	İstanbul	11.00 ± 1.0 ^a,b^	11.00 ± 1.0 ^a,b^	8.75 ± 0.8	10.00 ± 0.5 ^a,b^
Marmara	Kırklareli	13.50 ± 0.5 ^a,b^	11.00 ± 1.0 ^a,b^	10.00 ± 1.0 ^a,b^	12.50 ± 0.5 ^a^
Marmara	Kocaeli	12.25 ± 0.2 ^a,b^	10.50 ± 0.5 ^a,b^	10.75 ± 0.8 ^a,b^	9.50 ± 0.5 ^a,b^
Marmara	Tekirdağ	11.50 ± 0.5 ^a,b^	10.50 ± 0.5 ^a,b^	14.00 ± 1.0 ^a^	10.50 ± 0.5 ^a,b^
Mediterranean	Adana	10.00 ± 1.0 ^a,b^	9.50 ± 0.5 ^a,b^	12.50 ± 0.5 ^a,b^	10.00 ± 0.0 ^a,b^
Mediterranean	Gaziantep	11.00 ± 1.0 ^a,b^	10.00 ± 0.5 ^a,b^	10.00 ± 1.0 ^a,b^	9.00 ± 0.0 ^b,c^
Mediterranean	Hatay	11.00 ± 1.0 ^a,b^	10.50 ± 0.5 ^a,b^	10.00 ± 0.0 ^a,b^	9.50 ± 0.5 ^a,b^
Mediterranean	Mersin	13.50 ± 0.5 ^a,b^	10.50 ± 0.5 ^a,b^	12.50 ± 0.5 ^a,b^	9.50 ± 0.5 ^a,b^
	Mean values	11.40	10.28	10.58	9.82
	Negative Control **	0	0	0	0
	Positive Control ***	50	35	30	30

Significant differences in the same column are represented by different letters (a–c) (*p* < 0.05). ** 10% dimethyl sulfoxide (DMSO) solution was used. *** For bacteria, ampicillin (10 μg-Oxoid) commercial discs were used. *** For mold and yeast, vorozanole (11 μg) commercial discs were used.

## Data Availability

Data are contained within the article.
